# Catheter based intraperitoneal chemotherapy using taxane for peritoneal metastases from gastric cancer: from bench to bedside—a narrative review

**DOI:** 10.1007/s10120-026-01767-2

**Published:** 2026-06-18

**Authors:** Joji Kitayama

**Affiliations:** Department of Surgical Oncology, Japan Institute for Health Security, Toyama 1-21-1, Shinjuku, Tokyo, 162-8655 Japan

**Keywords:** Peritoneal metastasis, Peritoneal–plasma barrier, Catheter-based intraperitoneal chemotherapy, Paclitaxel, Docetaxel, Conversion surgery

## Abstract

Peritoneal metastasis (PM) is the most common and clinically devastating mode of spread in Gastric Cancer. Despite advances in systemic therapy, outcomes for gastric cancer with peritoneal metastasis (GCPM) remain poor, largely because the distinctive biology of PM, including sparse vascularization and the peritoneal–plasma barrier, restricts drug delivery and promotes therapeutic resistance. To overcome these limitations, locoregional approaches such as cytoreductive surgery plus hyperthermic intraperitoneal chemotherapy (CRS + HIPEC) and pressurized intraperitoneal aerosol chemotherapy (PIPAC) have been introduced, but their broader use remains limited by invasiveness, patient selection challenges, and variable survival benefits. In this setting, catheter-based normothermic intraperitoneal chemotherapy (CBIP) has emerged as a practical and scalable strategy, particularly in East Asia. By enabling repeated intraperitoneal administration through an implanted port, CBIP provides sustained regional drug exposure with minimal procedural burden and can be readily combined with systemic therapy. Among available agents, Paclitaxel and Docetaxel are especially suitable because of their prolonged peritoneal retention and low systemic absorption. Accordingly, taxane-based CBIP represents a biologically rational and clinically feasible platform for GCPM. In this review, we summarize its pharmacologic basis, clinical evidence, and future innovations in drug formulation and delivery that may further improve outcomes in this highly challenging disease.

## Introduction

Gastric cancer (GC) remains a major global health burden, ranking as the fifth most commonly diagnosed malignancy and the fifth leading cause of cancer-related death, with nearly one million new cases and approximately 650,000 deaths each year. The incidence is particularly high in East Asia, Eastern Europe, and South America [1,2]. Peritoneal metastasis (PM) represents the predominant metastatic pattern in GC, occurring in approximately 40–50% of patients with stage IV disease in population-based analyses [3–6]. This frequency is especially high in Bormann Type IV, diffuse-type, poorly cohesive, and signet-ring cell carcinomas [7, 8]. Notably, even after curative gastrectomy followed by contemporary adjuvant chemotherapy, the peritoneum remains the most frequent site of relapse, accounting for 40–50% of recurrences and rising to 50–60% in patients with serosa-positive tumors [9, 10]. Achieving effective control of PM is therefore pivotal to improving outcomes in patients with advanced GC.

Systemic chemotherapy remains the standard treatment for patients with gastric cancer with peritoneal metastasis (GCPM), similar to its use in other forms of metastatic disease [11, 12]. However, PM is biologically distinct from other metastatic patterns, characterized by very unique pathophysiological features, such as sparse vascularization [13], dense stromal fibrosis [14], the peritoneal–plasma barrier (PPB) [15], and immunosuppressive microenvironment [16], all of which severely impair drug delivery and limit the efficacy of systemic therapy [17]. Consequently, median survival remains poor, typically ranging from 4 to 12 months with systemic chemotherapy alone [18–20]. Even in recent clinical trials using immune checkpoint inhibitors, their subclass analyses suggest that they are less effective in patients with PM compared with those without PM. [21, 22]. These observations underscore the urgent need for alternative strategies to enhance drug delivery to peritoneal lesions.

Intraperitoneal (IP) chemotherapy enables direct delivery of high concentrations of cytotoxic agents into the peritoneal cavity, providing a strong pharmacologic rationale for treating PM. Among available approaches, cytoreductive surgery (CRS) plus hyperthermic intraperitoneal chemotherapy (HIPEC) has been widely adopted, particularly in Western countries. By combining macroscopic tumor removal with heat-enhanced cytotoxicity and tissue penetration [23], HIPEC has shown benefit in selected patients, particularly in ovarian [24] and colorectal [25] cancers. However, its efficacy is highly dependent on tumor biology, completeness of cytoreduction, and careful patient selection [26, 27]. In GCPM, benefit appears confined to highly selected patients [28, 29], and recent randomized trials have questioned its survival advantage [30, 31]. Pressurized intraperitoneal aerosol chemotherapy (PIPAC), developed mainly in Europe, uses aerosolized chemotherapy under pressure to improve intraperitoneal distribution and tissue penetration [32, 33]. Early studies in GCPM have shown encouraging survival, pathological responses, and occasional conversion to resectability, with favorable safety and quality-of-life (QOL) profiles [34–38]. Nevertheless, the absence of controlled trials still remains a major limitation.

Another practical strategy is normothermic IP chemotherapy delivered repeatedly through a subcutaneous port and intraperitoneal catheter. This approach allows sustained regional therapy without repeated surgery and can be readily combined with systemic chemotherapy. In Western countries, this approach has been primarily evaluated in ovarian cancer, demonstrating favorable clinical outcomes [39, 40]. In Japan and other Asian countries, it has been adapted for GCPM, and was historically termed neoadjuvant intraperitoneal and systemic chemotherapy (NIPS) since several cases subsequently underwent CRS [41, 42]. However, because such treatment setting should in reality be considered as advanced/metastatic rather than neoadjuvant, we refer to this modality as catheter-based normothermic intraperitoneal chemotherapy (CBIP). In this review, we summarize the experimental rationale, clinical evidence, and emerging innovations that may further enhance its therapeutic impact in GCPM.

## Mechanistic insights and implications for intraperitoneal (IP) chemotherapy: peritoneal–plasma barrier (PPB)

The peritoneal cavity constitutes the largest enclosed compartment in the human body, lined by a mesothelial monolayer and characterized by abundant visceral adipose tissue and a distinct local immune microenvironment. Despite its extensive surface area, effective peritoneal blood flow is remarkably limited and is estimated to be only 60–100 mL/min (approximately 1–2% of cardiac output): a feature that fundamentally distinguishes it from other visceral organs and contributes to the limited efficacy of systemic chemotherapy in peritoneal malignancies [13, 43].

More importantly, the peritoneal–plasma barrier (PPB) represents a critical determinant of pharmacokinetics and pharmacodynamics within the peritoneal cavity [15, 44]. The PPB is not a single structure but a dynamic, multilayered interface encompassing the mesothelium, submesothelial interstitium, and subperitoneal microvasculature. Solute transport across this barrier, via diffusion through the interstitium and absorption into capillaries, underlies the characteristic pharmacokinetic advantage of IP administration, enabling high local drug concentrations and prolonged residence times relative to intravenous delivery. Classical studies have identified the microvascular endothelium as the principal rate-limiting component, with transport kinetics strongly influenced by physicochemical properties such as molecular weight, charge, and solubility [44, 45]. Accordingly, larger molecules exhibit slower clearance and higher peritoneal-to-plasma area under the concentration–time curve curve (AUC) ratios [46, 47].

Beyond vascular factors, the submesothelial interstitium, particularly extracellular matrix composition and collagen density, has emerged as a critical regulator of solute diffusion [48, 49]. More recently, the endothelial glycocalyx, including hyaluronan, has been implicated in selectively modulating macromolecular permeability [50]. These findings collectively underscore that the PPB is a highly dynamic interface, continuously reshaped by physiological and pathological processes. Inflammation and tumor infiltration induce angiogenesis, fibrosis, and mesothelial disruption, profoundly altering transport properties and drug distribution.

Importantly, the PPB functions bidirectionally. While it restricts systemic absorption of IP-administered agents, thereby preserving high intraperitoneal drug exposure, it also limits drug delivery from the systemic circulation into peritoneal lesions. It preserves high intraperitoneal drug exposure by limiting systemic absorption of IP agents, yet simultaneously restricts delivery of systemically administered drugs into peritoneal lesions. This obstacle is further intensified by tumor fibrosis and desmoplasia, which raise interstitial density and pressure, creating a powerful barrier to diffusion not only from the bloodstream but also from the peritoneal cavity. Accordingly, the key therapeutic challenge is not simply achieving high drug concentrations at peritoneal surface but driving drugs deeply into metastatic nodules.

## Rationale of combination with systemic chemotherapy with CBIP-taxane

Among available chemotherapeutic agents, taxanes possess unique pharmacologic properties that make them particularly suitable for intraperitoneal (IP) administration. Paclitaxel (PTX) is highly hydrophobic and requires solubilization with vehicles such as Cremophor EL and ethanol, resulting in the formation of micellar particles with a relatively large molecular diameter (approximately 10–12 nm). These micelles are absorbed predominantly through the lymphatic system, leading to prolonged retention within the peritoneal cavity and limited systemic exposure [51]. Accordingly, after IP administration, the peritoneal-to-plasma area under the concentration–time curve (AUC) ratio for PTX has been reported to be approximately 1000 [52], with more recent studies showing a range of 550–2300, substantially higher than that observed with many hydrophilic agents [53, 54]. Docetaxel (DTX), another widely used taxane formulated with polysorbate 80 and ethanol, exhibits similar pharmacokinetic behavior, with prolonged peritoneal retention and reported peritoneal-to-plasma AUC ratios of 150–500 [54].

In addition to these pharmacokinetic advantages, PTX exerts antifibrotic effects, including inhibition of fibroblast proliferation and extracellular matrix deposition [55, 56], supporting its suitability for repeated IP administration. Consistently, preclinical studies have demonstrated that IP PTX achieves sustained locoregional retention in the cavity, high exposure of peritoneal tissues and direct penetration into peritoneal metastatic nodules [57–59].

**Fig. 1:**
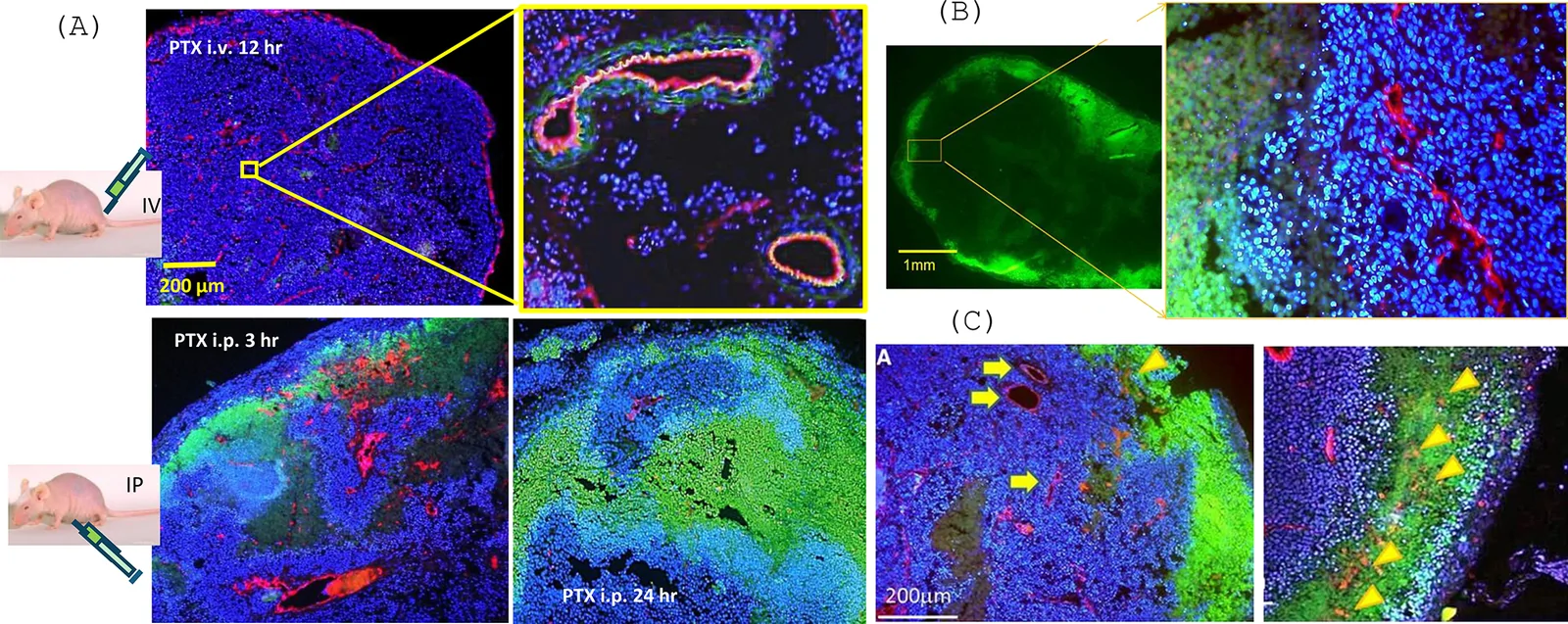
Peritoneal tumors were established by intraperitoneal (IP) inoculation of the human gastric cancer cell line MKN45P. Oregon Green–conjugated PTX (OG-PTX; 100 μg) was administered via the tail vein (intravenous [IV]; upper panels) or intraperitoneally (IP; lower panels). Tumors were harvested at the indicated time points and processed for frozen sections. Tumor vasculature was visualized by immunostaining for platelet/endothelial cell adhesion molecule-1 (PECAM-1/CD31) using an Alexa Fluor 594–conjugated secondary antibody, and nuclei were counterstained with DAPI. **A** Following IV administration, only small amounts of PTX distribution was observed around the intratumor vessels. In contrast, IP administration resulted in substantially higher PTX accumulation in the peripheral regions of peritoneal tumors. PTX penetrated up to several hundred micrometers from the tumor surface and accumulated predominantly in the avascular regions. **B**, **C** This peripheral accumulation was associated with induction of apoptosis in tumor cells (**B**) as well as peripheral vasculature (**C**). In **C** arrows indicate intact vessels, while arrowheads indicate damaged vessels

However, despite achieving high intraperitoneal concentrations, tumor penetration remains limited. Experimental studies using radiolabeled PTX and DTX have demonstrated that drug infiltration is limited to less than 100 μm from the tumor surface in xenograft models [60]. Accurate determination of drug penetration into peritoneal tumors remains technically challenging. Earlier studies suggested that some agents used during HIPEC may penetrate up to 2–5 mm; however, these estimates were largely based on pharmacokinetic modeling rather than direct microscopic evaluation [61]. Using fluorescence-based tissue analysis with Doxorubicin (DOX), one of the most reliable methods for assessing intratumoral penetration, DOX was shown to penetrate only a few cell layers (approximately 20–30 μm) after HIPEC [62]. In contrast, after PIPAC, DOX penetration reached 300–469 μm in ex vivo swine models [63, 64] and up to 500 μm in human patients [65]. Similarly, studies using fluorescein-labeled PTX demonstrated penetration of several hundred micrometers beneath the surface of peritoneal nodules (Fig. 1A, B), comparable to that reported for DOX after PIPAC [66, 67]. Importantly, because the findings were obtained after a single IP administration of PTX, repeated injection may allow deeper intratumoral penetration.

**Fig. 2:**
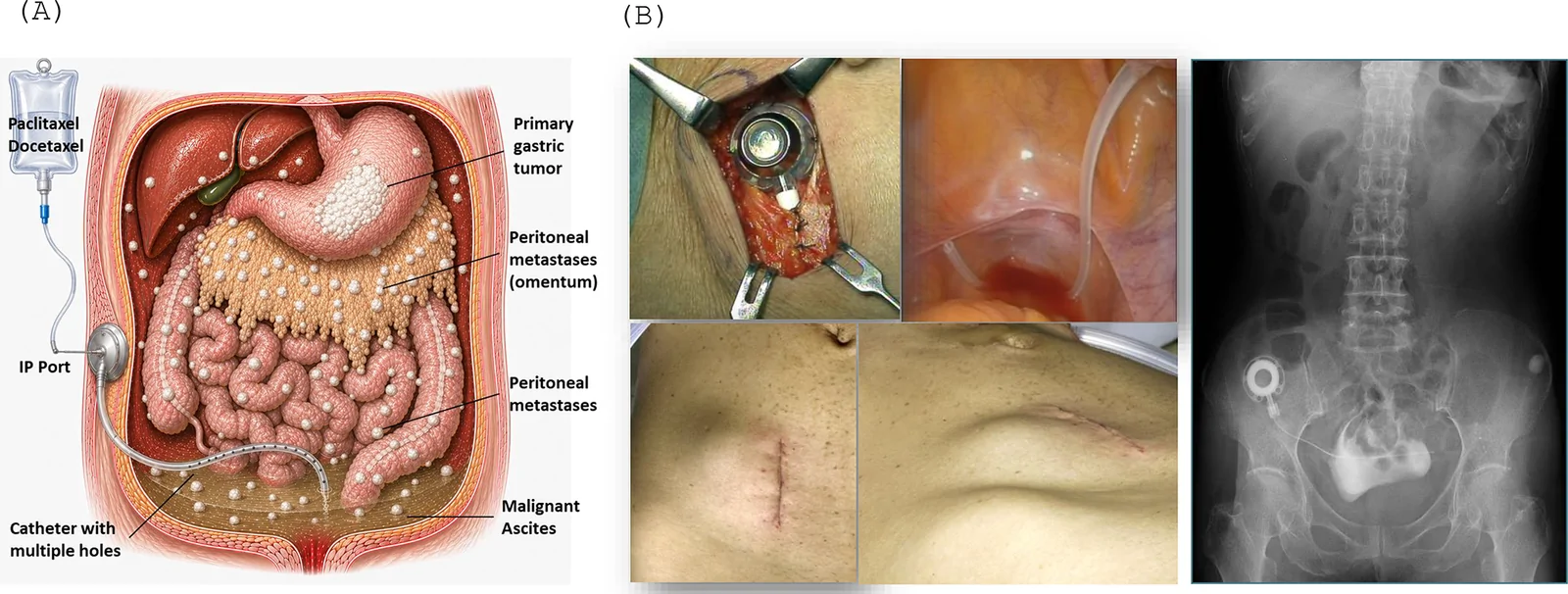
Catheter-based intraperitoneal chemotherapy (CBIP) using an implantable port system. **A** Schematic diagram **B** Photographs and abdominal X-rays of a patient with an IP port implanted

While this superficial distribution is sufficient to induce marked cytotoxicity, including destruction of tumor cells in the peripheral zone, it is unlikely to eradicate deeply located tumor cells. Consequently, single-dose administration is unlikely to achieve durable disease control, particularly in bulky or deeply invasive lesions. Repeated IP administration may partially overcome this limitation by enabling cumulative drug penetration. In this context, CBIP provides a practical platform for sustained and repeated IP drug delivery without the need for repeated surgical intervention. In this method, a subcutaneous access port is implanted in the lower abdomen and connected to an intraperitoneal catheter, typically positioned in the pouch of Douglas. (Fig. 2A, B) Placement is performed under laparoscopy or laparotomy and is generally completed within approximately one hour with minimal operative morbidity. Once established, this system allows non-invasive, repeated, typically weekly, administration of high local concentrations of anticancer agents over prolonged periods with a low incidence of treatment-related complications [68]. In addition, peritoneal fluid can be readily obtained via the port prior to drug infusion, allowing for assessment of viable tumor cells and tumor-derived components within the peritoneal cavity. This capacity for frequent and sustained drug delivery represents a key advantage of CBIP over HIPEC and PIPAC, both of which require general anesthesia and are not amenable to repeated administration. Given the aggressive biology and rapid regrowth kinetics of GCPM, treatment frequency and duration of drug exposure are likely critical determinants of durable disease control.

Even after repeated IP administration, the penetration depth into PM is still limited. Furthermore, IP therapy exerts minimal impact on extra-peritoneal metastases including occult disease and the primary tumor. In addition, in patients with prior abdominal surgery, postoperative adhesions may hinder drug distribution by creating compartmentalized spaces that are inaccessible to IP delivered agents. These limitations provide a strong rationale for integration with systemic chemotherapy. Preclinical studies demonstrate complementary spatial distribution patterns: Intravenously (IV) administered PTX preferentially localize to perivascular regions, whereas IP-delivered PTX accumulate in relatively avascular compartments. (Fig. 1A) [66, 67] This complementary distribution pattern provides a strong rationale for bidirectional chemotherapy, which may achieve more homogeneous intratumoral drug exposure. In addition, PTX has been shown to induce marked destruction of tumor cells and microvessels, particularly in the tumor periphery. (Fig. 1A, C) [67, 69], which may disrupt tumor architecture and reduce interstitial fluid pressure, thereby facilitating deeper penetration of IV administered agents. Supporting this concept, recent experimental data show that IP PTX enhances the intratumoral concentration of IV administered Carboplatin [70], providing direct evidence for the synergistic potential of sequential IP and systemic chemotherapy. Collectively, these findings indicate that CBIP using taxane is an ideal therapeutic strategy to overcome spatial barriers and achieve effective drug delivery to peritoneal tumors.

## Clinical results of CBIP-taxane with systemic chemotherapy for patients with GCPM

### Survival results for established GCPM

**Fig. 3:**
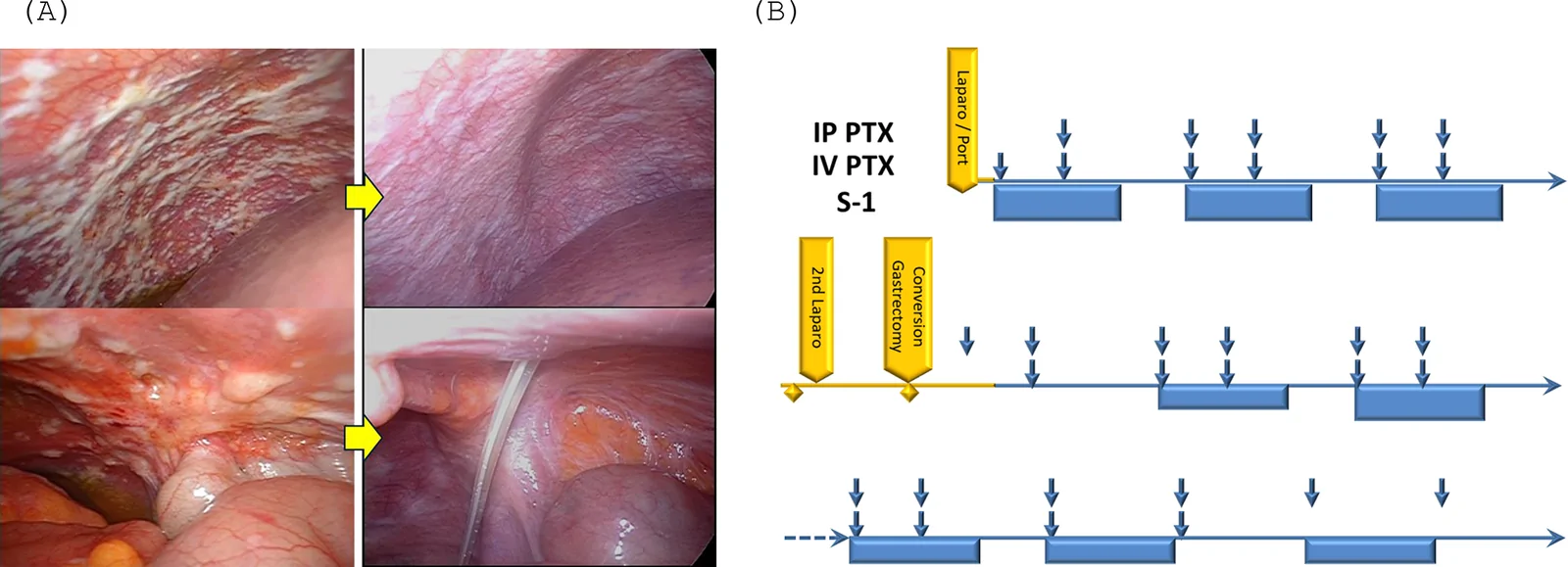
Laparoscopic views (**A**) and treatment course (**B**) of a representative patient with peritoneal metastasis from gastric cancer (GCPM) before and after CBIP using original regimen (S1+IV-PTX and IP-PTX) Cassell Militarr Paperbacks; 2002

Based on this background, patients with PM confirmed by staging laparoscopy were treated with CBIP using PTX in Japan. The initial regimen comprised oral S-1 (80 mg/m^2^, days 1–14, every 3 weeks), weekly IV PTX (50 mg/m^2^), and IP PTX administered on days 1 and 8 via an implanted peritoneal port. A phase I study established the recommended IP-PTX dose at 20 mg/m^2^ [71], and subsequent phase II trials in patients with macroscopic PM or positive peritoneal cytology demonstrated encouraging outcomes, including a 1-year overall survival rate (OS) of 78% and a median survival time (MST) of 22.5 months [72]. Comparable outcomes were reported in another phase II study involving 35 patients with macroscopic PM [73]. Notably, substantial regression of peritoneal lesions was frequently observed on second-look laparoscopy, enabling conversion gastrectomy in a subset of patients. In these cases, CBIP chemotherapy with PTX was continued postoperatively (Fig. 3A, B).

The two preceding phase II studies culminated in the phase III randomized PHOENIX-GC trial, designed to determine whether combined IP and IV PTX plus S-1 could improve survival compared with the standard cisplatin (CDDP) plus S-1 (SP) regimen [74]. A total of 183 patients were enrolled across 20 Japanese centers, of whom 164 were included in the full analysis set (FAS). In the primary analysis, MST was numerically longer in the IP group than in the SP group (17.7 vs. 15.2 months; hazard ratio [HR], 0.72), but the difference did not reach statistical significance (*p* = 0.08). Interpretation of these results is substantially limited by treatment refusal and crossovers to the IP chemotherapy outside the trial, which occurred in 12% of patients assigned to the SP arm. Unfortunately, study design was constrained by funding and by expectation of slower patient accrual, and the sample size had not been calculated to accommodate protocol violations of this magnitude. When restricted to the per-protocol population, the survival advantage of the IP regimen became significant (MST 17.7 vs. 14.3 months; HR, 0.64; *p* = 0.022), with a marked separation in long-term outcomes, as reflected by 3-year overall survival rates of 21.9% versus 6.0%. More importantly, the IP arm included a higher proportion of patients with ascites, representing a clinically unfavorable subgroup. Adjustment for this imbalance further amplified the treatment effect (HR, 0.59; *p* = 0.008), underscoring the robustness of the survival signal. Consistently, the IP regimen demonstrated superior control of peritoneal disease biology, with significantly higher rates of ascites resolution and cytological conversion (76% vs. 33%). Taken together, although PHOENIX-GC formally failed to meet its primary endpoint, the totality of evidence, including per-protocol and sensitivity analyses, provides a compelling signal of clinically meaningful benefit with IP-based therapy.

Several phase I/II studies have investigated the efficacy of IP-PTX in combination with various systemic chemotherapy regimens for GCPM, consistently demonstrating encouraging clinical outcomes (Table 1). In three studies evaluating IP-PTX (40 mg/m^2^) combined with S-1 (80 mg/m^2^ daily) and oxaliplatin (L-OHP, 100 mg/m^2^) (SOX regimen), 1-year overall survival (OS) rates ranged from 71 to 79% [75–77]. Comparable survival outcomes were observed with higher-dose IP-PTX (80 mg/m^2^) in combination with SOX [78, 79], suggesting that dose escalation does not necessarily translate into additional survival benefit within this regimen. Dose-finding phase I studies have reported variable optimal dosing of IP-PTX depending on the accompanying systemic therapy [71, 80–82]. Notably, higher doses of IP-PTX (60–80 mg/m^2^) have been shown to be feasible in combination with FOLFOX, with acceptable toxicity profiles and survival outcomes comparable to other regimens [81, 83]. In addition, Chia et al. reported that IP-PTX (40 mg/m^2^) combined with capecitabine and oxaliplatin achieved a MST of 14.6 months [84]. Similarly, Kobayashi et al. demonstrated that IP-PTX (20 mg/m^2^) combined with S-1 and cisplatin yielded a 1-year OS rate of 73.6% and an MST of 19.4 months [85]. Most recently, phase II and subsequent multicenter phase III (DRAGON-01) trial conducted in China provided further high-level evidence supporting the efficacy of this approach [86, 87]. In this study, IP-PTX combined with IV PTX and S-1 significantly improved OS compared with systemic therapy alone (MST 19.4 vs. 13.9 months, *p* = 0.0054), with sustained benefits observed in long-term survival outcomes, including 1-, 2-, 3-, and 5-year OS rates. Although the control arm does not fully represent current first-line systemic standards, combining IP-PTX with more contemporary intensive regimens, including molecularly targeted or immunotherapeutic agents, may further improve patient outcomes. In this context, the phase II Dragon-09 trial evaluating sintilimab (anti-PD-1 antibody) plus S-1 in combination with IV- and IP-PTX is currently underway in China [88].

**Table 1:** Efficacy of Catheter-based intraperitoneal chemotherapy (CBIP) using taxane for Patients with GCPM

Author, Year	IP regimen	Systemic regimen	Study	n	MST	1y-OS	Response rate	Cytology negative conversion rate	conversion Surgery rate	References
*Paclitaxel*										
Ishigami H, 2010	PTX (20 mg/m^2^)	S-1 + PTX	P2	40	22.5 M	78.0%	10/18 (55.6%)	86.0%	37.5%	[72]
Yamaguchi H, 2013	PTX (20 mg/m^2^)	S-1 + PTX	P2	35	17.6 M	77.1%	5/7 (71.4%)	97.0%	31.4%	[73]
Ishigami H, 2018	PTX (20 mg/m^2^)	S-1 + PTX	P3	114	17.7 M	71.9%	9/17 (52.9%)	95.0%	45.7%	[74]
Fujiwara, 2016	PTX (40 mg/m^2^)	S-1 + L-OHP	P2	60	ND	71.5%	4/6 (67.7%)	71.0%	35..9%	[75]
Shi M, 2021	PTX (40 mg/m^2^)	S-1 + L-OHP	P2	30	15.3 M	ND	ND	ND	36.7%	[77]
Saito S, 2021	PTX (40 mg/m^2^)	S-1 + L-OHP	P2	44	25.8 M	79.5%	2/3(67.7%)	86.0%	45.0%	[76]
Vatandoust, S, 2022	PTX (10 ~ 30 mg/m^2^)	Cap + CDDP	P1	15	11.5 M	46.7%	ND	ND	ND	[80]
Kang, SH, 2022	PTX (40 ~ 100 mg/m^2^)	mFOLFOX	P1	13	16.6 M	ND	3/6(50%)	80.0%	30.8%	[81]
Yang ZY, 2022	PTX (20 mg/m^2^)	S-1 + PTX	P2	67	19.3 M	67.2%	22/64 (34.4%)	67.2%	62.9%	[86]
Zhao S, 2022	PTX (80 mg/m^2^)	mFOLFOX	P2	29	11.0 M	ND	11/29 (37.9%)	ND	ND	[83]
Chia, DKA,2023	PTX (40 mg/m^2^)	Cap + L-OHP	P2	44	14.6 M	67.8%	ND	70.5%	29.5%	[84]
Tu, L, 2023	PTX (80 mg/m^2^)	S-1 + L-OHP	P2	49	16.9 M	81.6%	21/47 (51.2%)	ND	26.5%	[79]
Kobayashi D, 2024	PTX (20 mg/m^2^)	S1 + CDDP	P2	53	19.4 M	73.6%	ND	64.0%	30.0%	[85]
Seo WL, 2025	PTX (80 mg/m^2^)	S-1 + L-OHP	P2	28	NR	76.9%	10/24 (41.7%)	ND	ND	[78]
Badgwell B	PTX (100 mg/m^2^)	S1	P1/2	25	18.8 M	84.0%	ND	ND	ND	[82]
Yan C	PTX (20 mg/m^2^)	S-1 + PTX	P3	148	17.7 M	69.6%	ND	ND	50.7%	[87]
*Docetaxel*										
Fushida S, 2013	DTX(45 mg/m^2^)	S-1	P1/2	39	16.2 M	70.4%	6/11(52%)	81.0%	35.9%	[90]
Fukushima R, 2017	DTX (10 mg/m^2^)	Cap + CDDP	P2	48	ND	75.0%	1/3 (33%)	76.0%	41.7%	[91]
Cho 2017	DTX(100 mg/m^2^)	Cap + CDDP	P1/2	39	15.1 M	ND	3/5 (60%)	ND	ND	[92]
Bin, Y, 2022	DTX (30 mg/m^2^)	S1 + L-OHP	P2	39	11.7 M	ND	19/39 (48.7%)	69.4%	ND	[93]

PTX, paclitaxel; DTX, docetaxel; L-OHP, oxaliplatin; CDDP, cisplatin; Cap, Capecitabine; ND, not described

In Western populations, clinical evidence remains limited but is steadily emerging (Table 3). In the United States, the prospective phase II STOPGAP study [89], evaluating IP-PTX in combination with 5-fluorouracil and leucovorin, has been completed and has subsequently progressed to a phase III trial (STOPGAP II). (https://clinicaltrials.gov/study/NCT07001748) In Europe, a phase III study, IPa-Gastric trial, is currently evaluating the added benefit of IP-PTX to standard fluoropyrimidine- and oxaliplatin-based systemic regimens. (https://clinicaltrials.gov/study/NCT07304271) These studies are expected to clarify the generalizability of IP taxane–based strategies beyond East Asian populations and to better define their role within global treatment paradigms.

In parallel, intraperitoneal docetaxel (DTX) has also been investigated in phase I/II settings, typically in combination with S-1 or capecitabine-based systemic chemotherapy. Despite variability in dosing (10–100 mg/m^2^), these studies have reported survival outcomes broadly comparable to those observed with IP-PTX, with MST ranging from 11.7 to 16.2 months and 1-year OS rates of approximately 70–75% [90–93]. These findings suggest that the therapeutic benefit may reflect a class effect of taxane-based intraperitoneal therapy rather than a drug-specific phenomenon. Overall, accumulating evidence from multiple prospective studies support CBIP chemotherapy using PTX or DTX as a promising therapeutic option for GCPM, particularly in achieving local disease control and enabling conversion strategies.

### Significance of conversion surgery

Beyond survival outcomes, IP-taxane demonstrates notable activity in controlling peritoneal tumors. Across studies, cytological conversion rates from positive to negative range from 64 to 97%, substantially exceeding those reported with systemic chemotherapy alone [72–76, 81, 84–86]. Objective response rates, although difficult to assess due to the paucity of measurable lesions, have been reported in the range of 33% to 71% [72–75, 78, 79, 81, 83, 86]. Importantly, this disease control has translated into a meaningful proportion of patients becoming eligible for conversion gastrectomy, with reported rates ranging from 26 to 63% [72–77, 79, 81, 84–87]. Although surgical indications varied substantially across studies, patients who underwent conversion surgery consistently achieved prolonged survival, with reported median survival times of 24.2 to 42.1 months in selected cohorts [72–74, 79, 84, 85, 87]. These findings suggest that IP-PTX may play an important role in enabling curative-intent strategies and achieving long-term survival in selected patients. However, the precise contribution of conversion gastrectomy remains uncertain because of substantial heterogeneity in disease burden, treatment response, and operative criteria among studies. Notably, carcinoembryonic antigen (CEA) mRNA levels in peritoneal fluid have been reported as a potential biomarker for predicting benefit from conversion gastrectomy [94]. Further studies are warranted to refine patient selection, determine the optimal timing of surgery, and establish the most effective surgical strategy.

### Toxicity profiles

Major grade 3/4 toxicities were summarized in Table 2. The most common toxicity associated with CBIP-taxane is hematologic toxicity resulting from bone marrow suppression. Previous studies have reported grade 3/4 leukocytopenia and neutropenia in 5.1–27.0% and 7.4–50.0% of patients, respectively. In contrast, anemia and thrombocytopenia are less frequent, with incidences ranging from 0–31.3% to 0–20.0%, respectively. Febrile neutropenia has been reported in 0–23% of cases. Importantly, most hematologic toxicities are manageable with appropriate dose modifications or temporary treatment interruptions. Non-hematologic toxicities, including sensory neuropathy, gastrointestinal disturbances, fatigue, and hepatic or renal dysfunction, have also been reported but generally occur at relatively low frequencies. Notably, abdominal pain, a potential concern with IP chemotherapy, is infrequently observed. Overall, both the incidence and severity of these toxicities appear comparable to those associated with conventional systemic chemotherapy.

**Table 2:** Major toxicity (Grade 3/4) of CBIP chemotherapy using taxane

Author, Year	IP regimen	Systemic regimen	Study	n	Hematological Toxicity				Major non-hematological Toxicity	Port-relted Toxicity					Refrences
					Leukocyte-penia	neutro-penia	anemia	Thrombo-cytopenia		Infection		Obstraction	others		
*Paclitaxel*															
Ishigami H, 2010	PTX (20 mg/m^2^)	S-1 + PTX	P2	40	18%	38%	10%	0%	Nausea vomit: 8%, Anorexa:5%	0%		2.5%			[72]
Yamaguchi H, 2013	PTX (20 mg/m^2^)	S-1 + PTX	P2	35	23%	34%	9%	0%	Nausea vomit:3%	0%		0%			[73]
Ishigami H, 2016	PTX (20 mg/m^2^)	S-1 + PTX	P3	114	25%	50%	14%	0%	Nausea vomit: 10%, Anorexia 10%, Febrile neutropenia 8%	2.6%		2.6%	Subcutaneous hematoma 0.8%, Intestinal fistula 0.8%		[74]
Fujiwara, 2016	PTX (40 mg/m^2^)	S-1 + L-OHP	P2	60	27%	50%	18%	10%	Anorexia 12%, Febrile neutropenia 6%	1.7%		1.7%			[75]
Shi M, 2021	PTX (40 mg/m^2^)	S-1 + L-OHP	P2	30	23.3%	23.3%	16.7%	20.0%	Sensory neuropathy 40%. Nausea vomit20%, Diarrea 26.7%,	0%		0%			[77]
Saito S, 2021	PTX (40 mg/m^2^)	S-1 + L-OHP	P2	44	11%	39%	14%	2%	Febrile neutropenia 5%	6.8%		6.8%			[76]
Yang ZY, 2022	PTX (20 mg/m^2^)	S-1 + PTX	P2	67	19.4%	26.9%	31.3%	6.0%	Liver dysfunction 9%	11.9%			Subcutaneous accumulation 11.9%		[86]
Zhao S, 2022	PTX (80 mg/m^2^)	FOLFOX	P2	29	13.8%	34.5%	3.4%	0.0%	Diarrhea20.7%						[83]
Chia, DKA,2023	PTX (40 mg/m^2^)	Cap + L-OHP	P2	44		18.0%	2.0%	2.0%	Febrile neutropenia 14%						[84]
Tu, L, 2023	PTX (80 mg/m^2^)	S-1 + L-OHP	P2	49	18.4%	40.8%	22.4%	6.1%	Nausea vomit: 22.5%, Diarrhea 12.2%,	0%		0%	0%		[79]
Kobayashi D, 2024	PTX (20 mg/m^2^)	S1 + CDDP	P2	53	8.0%	24.0%	30.0%	4.0%	Diarrhea 13%, Anorexia 17%	1.9%		1.9%	dislocation1.9%,		[85]
Seo WL, 2025	PTX (80 mg/m^2^)	S-1 + L-OHP	P2	28	20.8%	41.7%	0%	8.3%		12.5%				catheter split1.9%	[78]
Badgwell B	PTX (100 mg/m^2^)	S1	P1/2	25	16.0%	32.0%				ND		ND	ND		[82]
Yan C	PTX (20 mg/m^2^)	S-1 + PTX	P3	148	21.7%	19.9%	11.6%	5.1%	Fatigue 7.7%,Anorexia 5.1%	2.7%		2.0%	port rotation 0.7% wound dihescence 0.7%, subcutaneous metastasis 0.7%, reflux/subcutaneous accumulation 4.1%		[87]
*Docetaxel*															
Fushida S, 2013	DTX (45 mg/m^2^)	S-1	P1/2	39	7.4%	7.4%	0%	0%	Anorexia 18.5%	2.6%		0%			[90]
Fukushima R, 2017	DTX (10 mg/m^2^)	Cap + CDDP	P2	48	8.0%	21.0%	29.0%	6.0%	Nausea vomit: 17%, Anorexia 25%,	4.2%		2.1%			[91]
Cho 2017	DTX (100 mg/m^2^)	Cap + CDDP	P1/2	39	5.1%	38.6%	25.6%	0%	Sensory neuropathy 10.3%. Mucositis 10.3%, Anorexia 12.8%, Fatigue 20.5%	5.1%					[92]
Bin, Y, 2022	DTX (30 mg/m^2^)	S1 + L-OHP	P2	39	20.0%	26.0%	13.0%	2.0%	Nause vomit 10%	ND		ND	ND		[93]

PTX, paclitaxel; DTX, docetaxel; L-OHP, oxaliplatin; CDDP, cisplatin; Cap, Capecitabine; ND, not described

An additional toxicity specific to CBIP is complications related to the intraperitoneal port. In a cohort study from the University of Tokyo, 20.6% of patients experienced port-related complications, including inflow obstruction (7.6%), infection (6.9%), reflux (3.1%), subcutaneous masses (1.5%), and intestinal or vaginal fistula (1.5%)[95]. However, complications requiring surgical intervention were relatively uncommon. Consistent with this, phase II and III studies have reported low rates of grade 3/4 port-related infection (0–4.2%) and obstruction (0–6.8%). Collectively, these findings indicate that the toxicity profile of CBIP is manageable and generally favorable, particularly when compared with more invasive approaches such as HIPEC or PIPAC and can be tolerated even in patients with relatively poor performance status.

### Clinical effects for patients with “microscopic” peritoneal metastasis

Positive peritoneal cytology (CY1) is classified as stage IV disease associated with a poor prognosis even in the absence of macroscopic metastases [96–98]. Given the demonstrated efficacy of IP-PTX in inducing regression of macroscopic peritoneal lesions, it is biologically plausible that this approach may also eradicate microscopic or minimal residual disease. However, clinical evidence in this specific population remains limited. Some promising results have been reported in patients with P0CY1 disease. Aizawa et al. analyzed 39 patients treated with a combination of IP-PTX (20 mg/m^2^), S-1 (80 mg/day), and IV-PTX (50 mg/m^2^), achieving cytological conversion from CY1 to CY0 in 95% of cases and a 1-year OS rate of 84.2% [99]. Similarly, Ding et al. reported outcomes in 36 patients treated with an intensified regimen incorporating apatinib, demonstrating cytological conversion rates of 77.8% and a 1-year OS rate of 80.6% [100]. Collectively, these findings suggest that IP-PTX–based therapy is highly effective in achieving cytological clearance, thereby potentially enabling conversion to curative-intent surgery in selected patients with initially microscopic peritoneal disease.

In addition, IP-PTX may have a powerful role in preventing peritoneal recurrence following curative resection of advanced GC, particularly in patients with serosal involvement. In Asia, S-1– or capecitabine-based regimens are standard adjuvant therapies for stage II/III disease; although these treatments reduce recurrence risk, peritoneal relapse still occurs in approximately 10.7–22.7% of patients, with higher rates observed in those with T3/4 tumors [9, 10, 101, 102]. Preliminary data suggest that IP-PTX may further reduce peritoneal recurrence in high-risk populations [103], and a phase III trial focusing on patients with type 4 GC is currently ongoing [104].

### Clinical effects for patients with highly advanced PM with massive ascites

Massive malignant ascites represents one of the most severe clinical manifestations of GCPM, frequently associated with poor performance status, impaired oral intake, and malnutrition. Consequently, such patients are often excluded from clinical trials, and disease-specific evidence in this population remains limited. In this setting, drug delivery from the systemic circulation is particularly compromised, resulting in poor clinical outcomes, with reported MST of only several months even now [105–107]. These limitations provide a strong rationale for intraperitoneal therapeutic approaches. Consistent with this, a subgroup analysis of the PHOENIX-GC trial demonstrated that the survival benefit of IP therapy became more pronounced with increasing ascites burden [74].

Cell-free and concentrated ascites reinfusion therapy (CART) is an apheresis-based modality in which ascitic fluid is filtered, concentrated, and reinfused intravenously, thereby restoring albumin and globulin levels. Originally developed in the 1970s, CART has been widely used in Japan as a palliative treatment for patients with refractory ascites [108, 109]. In addition to symptomatic relief, CART can notably improve performance status, thereby enabling the administration of subsequent systemic or regional therapies [110]. In this context, combination strategies incorporating CART and IP-PTX have been explored in Japan. A retrospective study of 30 GCPM patients with massive ascites treated with S1 + IV and IP-PTX in conjunction with CART reported a MST of 10.2 months and a 1-year OS rate of 43% [111]. Given the historically dismal prognosis of this population, these findings suggest that the integration of CART with CBIP-based therapy may represent a promising treatment strategy for patients with a high peritoneal tumor burden.

## Future improvement of CBIP-taxane

Based on its distinctive pharmacokinetic profile, CBIP taxane represents a theoretically well-founded strategy with supporting clinical evidence. However, intraperitoneal (IP) taxane therapy should be recognized fundamentally as a locoregional treatment directed at PM, with limited activity against extraperitoneal disease. Given that PM frequently coexists with systemic dissemination, the integration of effective and well-tolerated systemic chemotherapy is essential to optimize overall patient outcomes. In this context, combination strategies incorporating contemporary molecularly targeted agents and immunotherapies are particularly promising.

Recent phase II studies have incorporated IP-PTX into modern systemic regimens, thereby enabling the concurrent use of biomarker-driven therapies, including anti–PD-1 inhibitors and HER2-targeted agents (Table 3). Kang et al. reported outcomes in 22 patients treated with FOLFOX with or without nivolumab in combination with IP-PTX (60 mg/m^2^), demonstrating a MST of 20.2 months [112]. Yang et al. are currently investigating a combination regimen comprising cadonilimab (a dual CTLA-4/PD-1 inhibitor), LM-302 (a claudin 18.2–targeted antibody–drug conjugate), and S-1 with IP-PTX (20 mg/m^2^) in patients with CLDN18.2-positive gastric cancer (NCT06519591). In addition, a phase II trial (NCT05185947) is evaluating the combination of IP/IV PTX with the tyrosine kinase inhibitor nilotinib across multiple malignancies, including, but not limited to, gastric cancer [113]. These ongoing efforts are expected to refine the optimal systemic partners for IP-PTX–based therapy and to further enhance clinical outcomes through a rational, multimodal treatment approach.

**Table 3:** Major ongoing clinical trials on CBIP chemotherapy using taxane for GCPM

PI, responsible party	Trial name	Country	IP regimen	Systemic regimen	Study	Status	References
Senthil, M	STOPGAP	USA	PTX(40 mg)	5Fu + Leucovorin	P2	Completion	NCT04762953, Ref. 89
ECOG-ACRIN Cancer Research Group	STOPGAP II/EA2234	USA	PTX(40 mg)	5Fu + Leucovorin + PTX	P2/3	Recruiting	NCT07001748
Nilsson, M	IPa-Gastric	Sweden	PTX(40 mg)	Standard systemic regimens	P3	Recruiting	NCT07304271
Blakely, AM	ND	USA	PTX(20 ~ 60)mg)	Capacitabine, PTX + Nilotinib	P2	Completion	NCT04034251 Ref. 113
National University Hospital, Singapore	ND	Singapore	PTX(40 mg)	Capacitabine + Oxaliplatin + Immune checkpoint inhibitor or Trastuzumab	P2	Unknown	NCT01739894
Zhu, Z	Dragon-09	China	PTX(20 mg)	S1 + PTX + Sintilimab 200 mg	P2	Unknowm	NCT05204173
Yang, Z	Dragon-12	China	PTX(20 mg)	Cadonilimab, LM-302, and S-1	P2	Recruiting	NCT06519591
Kang, SH	IPLUS	Korea	PTX(60 mg)	mFOLFOX6 + nivolumab	P2	Completed	NCT03618758 Ref. 112

PTX; paclitaxel, 5Fu; 5-Fluorouracil

Another promising avenue for therapeutic advancement is the development of intraperitoneal (IP)–specific anticancer agents. A growing body of preclinical research has demonstrated that drug modification using advanced biomaterials, such as hydrogels, nanoparticles, and implantable delivery systems, enables sustained, locoregional drug release within the peritoneal cavity, thereby enhancing therapeutic exposure while minimizing systemic toxicity. These strategies and their translational potential have been comprehensively reviewed in recent literature [114, 115]. For PTX, both chemical modification and formulation-based approaches have consistently shown superior antitumor efficacy compared with conventional formulations in murine models. These improvements are largely attributable to enhanced tumor uptake, prolonged peritoneal retention, and favorable pharmacokinetic profiles [66, 116–121]. More advanced nanocarrier systems, including pH-sensitive polymersomes and tumor-penetrating peptide (iRGD)-functionalized nanoparticles, have further improved tumor accumulation and intratumoral penetration following IP administration [122, 123]. Comparable findings have also been reported for DTX in models of ovarian and colorectal peritoneal carcinomatosis, supporting the broader applicability of this approach across taxane-based therapies [124, 125].

Another important issue in CBIP is the impact on local immunity within the peritoneal cavity. The high drug concentrations achieved after IP-taxane may induce substantial cytotoxic effects not only on tumor cells but also on resident and infiltrating immune cells. Indeed, the peritoneal cavity harbors a highly distinctive immune microenvironment that is generally immunosuppressive and may critically influence the biological behavior and treatment responsiveness of PM [16, 126, 127]. A recent study demonstrated that, CD8(+) T cells were reduced, whereas CD11b(+) myeloid cells increased following IP PTX, and that expansion of CD16(−) CC chemokine receptor 3 (CCR3)(+) eosinophils was observed in a subset of patients and was associated with marked tumor regression and improved survival outcomes [128]. Given that the immune microenvironment is a key determinant of chemosensitivity [129]. reconstruction of effective antitumor immunity within the peritoneal cavity may be essential for maximizing the clinical efficacy of CBIP.

## Conclusion and future perspective

The usefulness of CBIP-taxane is increasingly supported by emerging clinical evidence. Recent Asian [130] and international [131] expert panels have suggested that catheter-based administration of PTX may be recommended in clinical practice, particularly in East Asian populations. Beyond its clinical and mechanistic validity, PTX also offers a major advantage in cost-effectiveness. While modern cancer therapies have improved outcomes, they have also increased “financial toxicity,” adversely affecting quality of life, treatment adherence, and survival [132, 133]. In contrast to costly molecular-targeted agents and immunotherapies, PTX is a widely available low-cost generic drug, making IP PTX a highly pragmatic and economically accessible treatment approach.

If cancer therapy is compared to modern “warfare”, PM may represent an impregnable “fortress” protected by the rigid “armor” of the PPB. Historical experience has demonstrated that relying solely on frontal attack against heavily fortified positions causes a major strategic failure associated with massive casualties [134]. By analogy, relying on systemic chemotherapy alone may result in similarly devastating outcomes. By contrast, the peritoneal cavity side may represent “Achilles’ heel” of this otherwise formidable “fortress”. By exploiting this unique anatomical and pharmacologic vulnerability, taxane-based CBIP has emerged as a strategically rational and promising treatment approach. Given the dismal prognosis of patients with GCPM, IP-PTX combined with optimal systemic chemotherapy should be considered as a reliable first line therapeutic option, simply for the benefit of the patients. The development of more effective IP-specific drugs that enable a dual-delivery paradigm is the most critical unmet challenge for achieving a true therapeutic breakthrough in the “battle” against GCPM.
